# Structural biology of presenilin 1 complexes

**DOI:** 10.1186/1750-1326-9-59

**Published:** 2014-12-18

**Authors:** Yi Li, Christopher Bohm, Roger Dodd, Fusheng Chen, Seema Qamar, Gerold Schmitt-Ulms, Paul E Fraser, Peter H St George-Hyslop

**Affiliations:** Cambridge Institute for Medical Research, Wellcome Trust MRC Building, Addenbrookes Hospital, Hills Road, Cambridge, CB2 0XY UK; Tanz Centre for Research in Neurodegenerative Diseases and Depts of Medicine, Laboratory Medicine and Pathobiology and Medical Biophysics, University of Toronto, Krembil Discovery Tower, 6th Floor- 6KD417, 60 Leonard Avenue, Toronto, Ontario M5T 2S8 Canada

**Keywords:** Presenilin, Nicastrin, APH1, PEN-2, Gamma-secretase, Epsilon secretase, Notch, APP, Abeta, Structural biology, Gamma-secretase inhibitor compounds, Gamma-secretase modulator compounds, Lateral gate

## Abstract

The presenilin genes were first identified as the site of missense mutations causing early onset autosomal dominant familial Alzheimer's disease. Subsequent work has shown that the presenilin proteins are the catalytic subunits of a hetero-tetrameric complex containing APH1, nicastrin and PEN-2. This complex (variously termed presenilin complex or gamma-secretase complex) performs an unusual type of proteolysis in which the transmembrane domains of Type I proteins are cleaved within the hydrophobic compartment of the membrane. This review describes some of the molecular and structural biology of this unusual enzyme complex. The presenilin complex is a bilobed structure. The head domain contains the ectodomain of nicastrin. The base domain contains a central cavity with a lateral cleft that likely provides the route for access of the substrate to the catalytic cavity within the centre of the base domain. There are reciprocal allosteric interactions between various sites in the complex that affect its function. For instance, binding of Compound E, a peptidomimetic inhibitor to the PS1 N-terminus, induces significant conformational changes that reduces substrate binding at the initial substrate docking site, and thus inhibits substrate cleavage. However, there is a reciprocal allosteric interaction between these sites such that prior binding of the substrate to the initial docking site paradoxically increases the binding of the Compound E peptidomimetic inhibitor. Such reciprocal interactions are likely to form the basis of a gating mechanism that underlies access of substrate to the catalytic site. An increasingly detailed understanding of the structural biology of the presenilin complex is an essential step towards rational design of substrate- and/or cleavage site-specific modulators of presenilin complex function.

## Introduction

**Figure 1:**
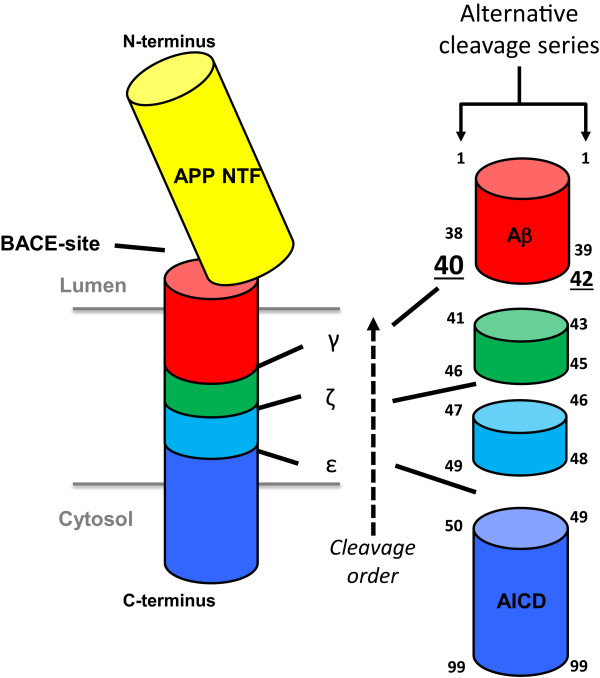
**Sequential cleavage sites on APP involved in the β-amyloid producing pathway.** Multiple species of Aβ can be produced. The most prevalent species end at residue 40, but species ending at residue 42, 38 etc. are also generated. Species ending at residue 42 are particularly prone to oligomerization.

Multiple lines of evidence suggest that the accumulation and aggregation/oligomerisation of the Aβ peptide plays a central role in the pathogenesis of Alzheimer’s disease (AD). Aβ is derived from the amyloid precursor protein (APP) after sequential cleavage of APP. In the first step of the amyloidogenic pathway, APP is cleaved by BACE1 to generate a soluble N-terminal fragment (β-sAPP) and a membrane-bound C-terminal fragment, C99. The C-terminal fragment is then cleaved through its transmembrane domain by the presenilin complex, thereby generating a series of proteolytic fragments that include Aβ peptides (released into the lumen) and amyloid intracellular domain (AICD, released into the cytosol) [1–8] (Figure 1). Aβ peptides so produced are of various lengths and differing abundance, but the principal species is Aβ40, with lesser amounts of Aβ42. The proteolytic cleavage of the C99 membrane-bound stub begins at the cytoplasmic face with the initial cleavage, termed ϵ-cleavage. The ϵ-cleavage occurs at residue 49 relative to the BACE cleavage site, just inside the inner membrane leaflet [3, 5, 6, 9–11]. A second set of cleavages occur at residue 46, termed, the ζ-cleavage site [12], producing Aβ46 plus a small labile C-terminal fragment [3, 13, 14]. The final cleavage occurs at the γ-cleavage site at residue 40, yielding Aβ40 (Figure 1). However, minor sets of cleavages give rise to other fragments, for instance Aβ48 (ϵ), Aβ45 (ζ) and Aβ42 (γ), which represent products of cleavages on the opposite face of the TM helix [11, 15–18]. Related ϵ- and γ- cleavage sites have been identified during Notch-1 cleavage, but are named as S3 and S4 cleavages, respectively [19].

Aβ peptides of different length have differing propensities to aggregate or to cause neurotoxicity [20]. Consequently, it has been proposed that therapeutic manipulation of Aβ neurotoxicity could be achieved either by inhibiting total Aβ production or by modulating the specific Aβ species produced [21–24]. Recent work with gamma-secretase modulator (GSM) compounds has highligted the difficulty in generating substrate-specific inhibitors that potently prevent the generation of amyloidogenic APP cleavage products but exhibit minimal activity toward the cleavage of other substrates such as Notch-1. Even semi-specific compounds, including semagacestat, inhibit the cleavage of non-APP target substrates to a degree that causes unacceptable side effects [25, 26]. This review examines the function of the presenilin complexes from a structural perspective and emphasizes aspects of their biology that will need to be understood before rational drug design approaches can be applied to achieve either improved substrate specificity and/or to modulate the species of Aβ produced.

### Presenilin complexes

**Figure 2:**
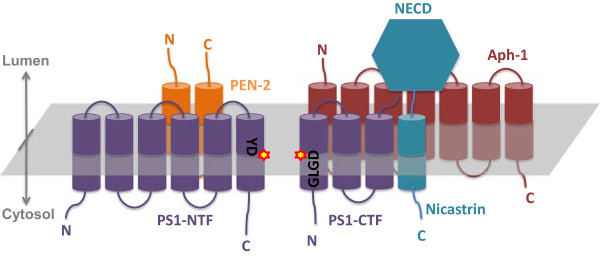
**Topology view of presenilin 1 complex subunits: presenilin (purple), nicastrin (blue), Aph1 (red) and PEN-2 (orange).** The highly conserved sequence in the catalytic pocket, YD287 and GLGD385 (presenilin 1 numbering), has been specified with stars.

The presenilin (PS) genes were first identified by this group during searches for genes responsible for early onset familial AD (FAD), [27, 28]. There are two presenilin genes in vertebrates: *PSEN1* (on chromosome 14, encodes PS1) and *PSEN2* (on chromosome 1, encodes PS2). Both PS1 and PS2 are ~50 kDa polytopic transmembrane proteins that interact with nicastrin, PEN-2 and APH1, to form the biologically active γ-secretase [29–35] (Figure 2). The assembly of these four components to a functional γ-secretase complex is tightly controlled and gives rise to a 1:1:1:1 heterotetrameric complex with a mass of 174 kDa, as determined by SEC-MALS [36]. γ-secretase complexes lacking any of their subunits are destabilised and degraded [37].

### Topology and structure of presenilin 1 complex components

#### Presenilin

PS1 (and PS2) are the catalytic subunits of the heterotetrameric presenilin 1 (or presenilin 2) complexes [29, 31] and are the archetypal members of the GXGD family of intramembranous aspartyl proteases, which also includes signal peptide peptidases (SPP) and a variety of archaeal homologues [38–42].

During the assembly and maturation of presenilin complexes, the PS1 or PS2 subunits undergo endoproteolytic cleavage into N- and C-terminal fragments [37, 43, 44]. For PS1 the cleavage occurs near residue Met298 (encoded by Exon9) within a cytoplasmic peptide loop encoded by Exon 8–10 [45].

Presenilin proteins adopt a membrane topology characterized by nine helical transmembrane (TM) domains. Once folded, its hydrophilic, flexible N-terminus is located in the cytosol. In contrast, its C-terminus is either embedded within the lumenal face of the lipid bilayer or protrudes into the lumen or extracellular space [46–49]. Crosslinking experiments using a series of TMD-swap PS1 mutants revealed that TM2 and TM6 are both in proximity to TM9 [50]. TM6 and TM7 each contain one of two aspartyl residues required for catalytic activity [31]. A solution state NMR structure of the PS1-CTF domain is available that was generated by using a cell-free expression system and SDS micelles for embedding the protein [51] (PDB code 2kr6). This model confirmed the prediction that TM7, TM8 and TM9 are likely to be helical and also revealed a small helix within the domain encoded by Exon 9. However, the results are difficult to interpret, given the solubilization of the protein fragment in SDS micelles, which may exert different constraints on protein folding than the lipid bilayer, and the absence of PS1-NTF and other presenilin subunits.

More recently, a crystal structure has been reported for a distant homologue of PS1 from the archaeon *Methanoculleus marisnigri* JR1 (MCMJR1) [52]. Like the human PS1 protein, this archaeal protein adopts a membrane topology that comprises nine transmembrane segments (TMs) and cleaves itself into N-terminal (encompassing TMs 1–6) and C-terminal (encompassing TMs 7–9) fragments, each of which contains a catalytic aspartate residue. The crystal structure of MCMJR1 reveals a protein fold in which the N-terminal domain wraps around the C-terminal domain positioned in the center of the structure. The two catalytic aspartate residues in the structure are observed not to be in close enough proximity in order for catalysis to occur. Indeed, the particular construct used in crystallisation, which has had a large section of the loop between TM6 and 7 proteolytically removed, is inactive (unpublished observations). The authors suggest a possible route for substrate access between TM6, TM7, and TM8. However, in the structural model presented the route appears to be almost entirely obstructed.

#### Nicastrin

Nicastrin was the first subunit of the complex to be cloned after the presenilins [35]. Nicastrin consists of a transmembrane helical domain and an extracellular glycosylated ectodomain. Nicastrin may be involved in regulating intracellular protein trafficking of the nascent presenilin complex during its assembly [53–55] and in binding to the N-terminus of substrates [56, 57]. Nicastrin associates with the hemicomplex comprising PS1-CTF and APH1 by binding to the the C-terminus of PS1 [58].

Nicastrin is a type I integral membrane protein and contains a conserved DYIGS motif that may be involved in substrate binding. The ectodomain of Nicastrin has been predicted to adopt an aminopeptidase/transferrin receptor-like secondary structure [59]. Indeed, due to considerable sequence similarities between the Nicastrin ectodomain (NECD) and both the human transferrin receptor (PDB code 1cx8) and the glutamate carboxyl peptidase PSMA (PDB code 2xef), the structure of the nicastrin ECD could be modelled using the X-ray crystal structures of these proteins as a template. This structural homology was recently confirmed in a cryo-EM study [60] (PDB 4upc) and by crystallography [61] (PDB 4r12). Prior to the atomic structures of NECD [60, 61], other structural studies had predicted additional structural domains downstream of the DYIGS motif and peptidase-like domains, near residue 571. This domain was initially predicted to be homologous to tetratricopeptide repeat (TPR) domains, which are commonly involved in peptide recognition [62]. However, a TPR fold was not apparent in the atomic structures of NECD, which contained most of this domain [60, 61].

The function of the NECD is currently the focus of some controversy. The NECD carries extensive glycosylation (potentially 16 sites, with ~36 kDa total mass) and adopts a thermostable structure [63]. The presence of non-functional peptidase domain in the NECD, together with observations that indicate nicastrin detects the lengths of extracellular N-terminal protrusions of substrate proteins, suggest it may be involved in substrate selection and acquisition [57]. However, nicastrin is not essential for γ-secretase activity [64].

#### PEN-2

PEN-2 and the fourth component of the complex, APH1, were both identified and cloned by genetic screens in invertebrates for enhancers and suppressors of Notch signaling [65] PEN-2 is a 101 residue (12 kDa) membrane protein with two predicted transmembrane domains. By introducing N-linked glycosylation sites into the N- and C-termini or the loop region between the two putative transmembrane helices of PEN-2, it has been possible to show that both termini of this protein are luminal, while the hydrophilic loop is cytosolic [66]. PEN-2 binds to the fourth transmembrane domain of PS1 [67–69] and helps to stabilise the γ-secretase complex after PS1 endoproteolysis [70]. PEN-2 is also suggested to play an essential but as yet poorly understood role in the final assembly step and activation of the mature complex [71].

#### APH1

Anterior Pharynx Defective 1 (APH1) is a protein of approximately 308 amino acids in *C. elegans* and 195–265 residues in mammals [65]. In humans, two paralogous genes, which map to Chromosomes 1 and 15, encode for the highly similar gene products APH1A and APH1B, respectively. A further duplication of the APH1B gene in mice gave rise to a third APH1 family gene, APH1C. Because only one copy of any of the available APH1 proteins is incorporated into a given presenilin complex, two different types of PS1 or PS2 complex are observed in humans, and three different types of PS1 or PS2 complex exist in mice [72]. The function of APH1 is still not well established, although it is clear that APH1 is required for γ-secretase activity. All human and mouse APH1 paralogs contain a conserved GXXXG motif that may be involved in interactions with other subunits in the presenilin complex [73]. The membrane topology of APH1 has been studied by selective permeabilization of the plasma membrane and immunofluorescence microscopy, which revealed that the protein is a multi-pass transmembrane protein with its C-terminus facing the cytosol. More detailed glycosylation mutagenesis experiments further revealed APH1 to acquire a seven-transmembrane topology with its N-terminus, as well as even-numbered loops, facing the lumen [74]. Several studies have shown that APH1 and nicastrin form a stable sub-complex [75, 76]. It has been suggested that the APH1:nicastrin complex forms an initial scaffold prior to the generation of the full presenilin complex [7, 8, 55, 77, 78]. As the assembly of the presenilin complex progresses the PS1-CTF subunit joins this initial scaffolding complex by an interaction between the extreme PS1 C-terminus and APH1 [58].

### Structure of presenilin complex: *early globular models*

Obtaining both static and dynamic structural models of the presenilin complex is an important step towards understanding how the complex works. Electron microscopy-based structural investigations of the presenilin complexes are non-routine and difficult due to its low molecular weight and lack of symmetry. Prior to 2014 [79–82], electron microscopy reconstruction studies generated a variety of 3D structure models differing in shape and volume. None of these were validated using independent biophysical methods. The first of these early models used negative stain electron microscopy to reveal a flat heart-shaped model resolved at 48 Å and exhibiting C2 symmetry [82]. The presenilin complex occupied a volume of 560 Å × 320 Å × 240 Å in this structural model. Afterwards, three more low resolution structure models were generated by negative stain or cryo-electron microscopy for PS1 complexes isolated in CHAPSO or digitonin [79–81]. The first of these was a 20 Å resolution globular structure model with a 120 Å diameter and a 20–40 Å-wide low density chamber. The model proposed openings at both the top and bottom surfaces [80] and attributed a small protrusion to represent the NECD. This model was then improved to a 12 Å cryo-electron microscopy model with a globular structure, dimensions of 80 Å × 90 Å × 85 Å and three solvent-accessible but non-perforating central cavities in the membrane-embedded domain [81]. The third globular model was based on cryo-EM data with 18 Å resolution, and depicted the presenilin complex with a cup-like shape and a lateral belt surrounding a water-accessible internal chamber. Based upon labeling experiments that made use of a γ-secretase transition state inhibitor coupled to gold particles, the catalytic site was thought to be located in this chamber. A structure model of a pre-activation, PEN-2-free complex was also built in this study. Comparisons between the pre-activation model and the model of the mature complex suggested that PEN-2 binding modifies the architecture of the active site during complex maturation [79].

### Structure of presenilin complex: *recent bi-lobed models*

**Figure 3:**
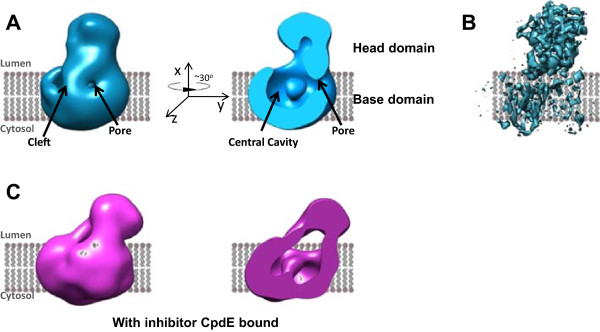
**Bi-lobed models of presenilin 1 complex by electron microscopy. (A)** The first bi-lobed structure model of PS1 complex, 14.7 using negative stain EM (EMD-2477). **(B)** The 4.5 Å model of PS1 complex by cryo-EM (EMD-2677), confirming its bi-lobed shape. **(C)** 14.7 Å model of PS1 complex bound with its non-transition state inhibitor Compound E (CpdE) (EMD-2478). This model revealed a conformational change induced by the inhibitor.

Early in 2014, a considerable advance in the structural modeling of the presenilin complex was achieved when negative stain 3D electron microscopy data were combined with several complementary cross-validating biochemical, pharmacological and biophysical methods including SEC-MALLS and FRET-FLIM [36]. The study provided an experimentally-validated structure and generated the first direct visualization of a structurally dynamic presenilin complex. Structures were built at 17 Å using single particle electron microscopic methods for both the native human PS1 complex and for the human PS1 complex following the binding of the non-transition state peptidomimetic γ-secretase inhibitor Compound E ((S,S)- 2-[2-(3,5-Difluorophenyl)-acetylamino]-N-(1-methyl-2-oxo-5-phenyl-2,3-dihydro-1H-benzo[e][1, 4]diazepin-3-yl)-propionamide, M.W. = 490.5 Da). In contrast to the globular shape predictions of earlier models, this work proposed that presenilin complexes have a bi-lobed shape, containing a larger base (93 Å × 93 Å × 60 Å) and a separate, smaller head (65 Å × 60 Å × 55 Å) (Figure 3A). The orientation of the complex was determined by immuno-labelling the N-terminus of nicastrin (residues 168–289), which demonstrated that the nicastrin ectodomain is located in the head domain of the bi-lobed complex (Figure 3A). In good agreement with this conclusion, the height of the base domain in this model is approximately 60 Å, which is sufficient to span the widths of most cellular membranes (35–40 Å) [83, 84] and, thus, large enough to contain the TM domains of PS1, PEN2, APH1, and NCT.

In partial agreement with some of the earlier models, this first bi-lobed model displayed a central cavity in the base domain that opens toward the lumenal/extracellular surface (Figure 3A). Crucially, the base domain contained a lateral cleft that communicated with the central cavity. This cleft was suggested to be part of a lateral gate mechanism involved in substrate access because it was closed by binding of Compound E, which blocks substrate binding to the Initial Substrate Docking site [36].

More recently, considerable advances in cryo-EM technology, in particular the use of new detectors and image processing methods, enabled further refinements of this model by increasing the image resolution to 4.5 Å [60] (EMD-2677, PDB code 4upc). This higher resolution model confirmed the bi-lobed shape of human presenilin complexes as their native state (Figures 2B and 3B). Multiple transmembrane helices were also visible in this new model but the resolution remained insufficient to assign individual TMs to specific subunits of the complex (Figure 3B).

### Subunit interaction and domain movement in the PS1 complex

#### Inhibitor-induced conformational change

The presence of certain detergents causes a concentration-dependent dissociation of the presenilin complex into two hemi-complexes [36, 85, 86]. Each hemicomplex contains one catalytic aspartate of the presenilin protein. One of the hemi-complexes consists of PS1-NTF and PEN2, while the other hemi complex consists of nicastrin, APH1 and PS1-CTF (Figure 2). Interestingly, inhibitors, such as Compound E, can stabilise the PS1 complex and prevent its detergent-induced dissociation [36]. This result suggests that inhibitor binding causes a conformational change that brings the complex components closer together. Such a conformational change would lead to the formation of new interactions between the hemi-complexes, resulting in an overall stabilisation of the complex.

This long-range conformational change in the complex was confirmed indirectly by *in vitro* intra-molecular Fluorescent Lifetime Imaging Microscopy – Förster Resonance Energy Transfer methods (FLIM-FRET) in which a donor GFP tag was added to the N-terminus of PS1-NTF and a acceptor RFP tag was cloned into the loop at the N-terminus of PS1-CTF. In the presence of Compound E, there was a significant change in FRET-FLIM, indicating that the two γ-secretase hemicomplexes have been brought closer together by Compound E binding [36].

These indirect experiments were then directly confirmed by negative stain single particle electron microscopy.

The EM structure model for Compound E-bound PS1 complexes was highly similar to the native complexes with a bi-lobed overall shape. However, there were several critical differences. Following binding of Compound E to a non-catalytic site on PS1-NTF, PS1 complexes undergo several allosteric conformational changes that include: 1) rotation of the nicastrin-containing head domain; and 2) compaction of the membrane embedded base domain with closure of the lateral cleft (Figure 3C) [36].

### Reciprocal cross-talk between the initial substrate docking site and the inhibitor binding site

Excitingly, the Li et al. study also revealed that there are several reciprocal long range interactions between the initial substrate docking site at the interface between the PS1-NTF and PS1-CTF and the Compound E binding site. Specifically, substrate docking *increases* inhibitor binding [36]. Conversely, Compound E binding induced a dose-dependent *reduction* in substrate binding.

These observations not only demonstrate that the presenilin complex is structurally dynamic, they show for the first time that there are important reciprocal long-range structural interactions occurring between different sites within the complex. These findings shed light on how non-catalytic site inhibitors might work (namely by allosterically closing the substrate docking site). Importantly, these findings also provide a testable hypothesis on how a series of reciprocal allosteric interactions could operate a lateral gate governing substrate access to the protected catalytic pocket. Thus, binding of substrate at the initial docking site might open a translocation pathway to permit movement of the substrate into the complex. Subsequent occupancy of sites (e.g. the site of binding by the peptide-mimetic Compound E inhibitor) in the translocation pathway might then close the initial docking site, until the substrate is cleaved. At that point, release of the reaction products relaxes the closure of the initial docking site and the enzyme can reconfigure to bind a new substrate molecule. Such a mechanism would account for the slow processivity of presenilin complexes.

### Structure of presenilin-like homologues

Signal peptide peptidases (SPP) form a family of intramembranous aspartyl proteases homologous to presenilins. The negative stain EM-based structure of human SPP was determined at 22 Å resolution [87]. The model revealed SPP as a slender, bullet-shaped homotetramer. Independent biochemical studies have also suggested that a homotetrameric complex may be the functional unit of SPP. The SPP EM model displayed a central chamber possibly analogous to the central chamber/cleft observed in the presenilin archeal homologues (see next paragraph) [87].

**Figure 4:**
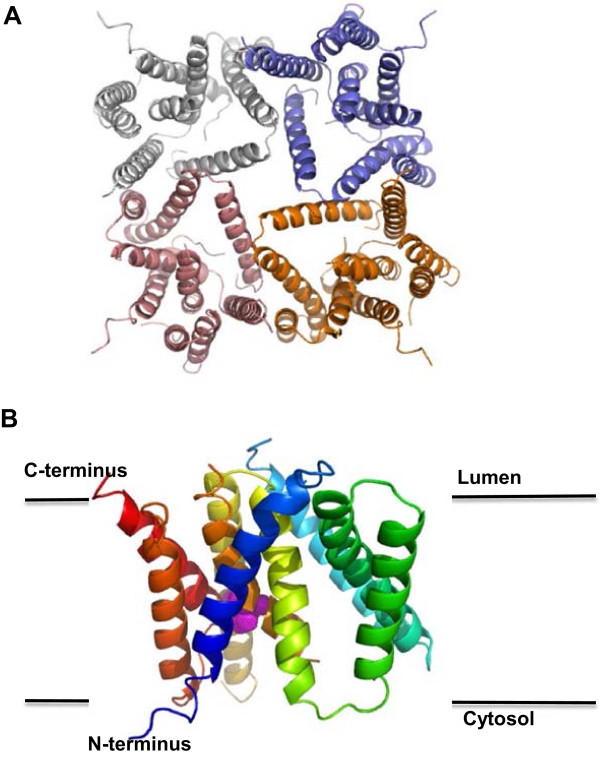
**Structure of presenilin protein homologue, SPP. (A)** Crystallographic tetramer of MCMJR1 (PDB 4hyc). The image is generated by PyMOL and coloured to emphasize different domains. **(B)** Rainbow coloured (The PyMOL Molecular Graphics System, LLC) MCMJR1 monomer structure (PDB 4hyc, chain A). The active site (magenta) is buried within a hydrophilic pocket between TM domains.

More recently, in 2013, a 3.3 Å crystal structure of the archeal GXGD presenilin-like aspartyl protease MCMJR1 (also known as mmPSH, PDB code 4hyc, 4hyd and 4hyg) (Figure 4A) revealed that the active site of MCMJR1 is buried in a hydrophilic pocket formed by the TM domains [52] (Figure 4B). A lateral cleft between TM6 (located on PS1-NTF in PS1) and TM9 (on PS1-CTF in PS1) and a central channel was proposed as a potential route for substrate access, although closer inspection reveals the cleft between TM6 and TM9 to be occluded in the crystal structure [52]. It is unclear whether this is: 1) an artefact of the mutagenesis required to get the protein to crystallise; 2) a packing artefact during crystallization; or 3) the correct structure. Whether the substrate accesses via a route between TM6 and TM9 as suggested by some crosslinking studies therefore remains unresolved.

## Conclusions

The recent structural studies on presenilin complexes represent a major advance toward the overall objective of understanding the molecular workings of the complex. The initial controversy surrounding the complex’s overall shape has been put to an end by the consistent documentation of a bi-lobed structure in the most recent models. This bi-lobed model was carefully validated by multiple independent methods and has since been further confirmed by higher resolution cryo-EM data. The head domain of the bi-lobed shape contains the NECD, and the base domain contains the transmembrane domains of all four subcomponent proteins. The head domain rotates when Compound E inhibitor is bound to the complex. In its native state the base domain adopts an “open” structure with a central cavity and lateral cleft opening to the side. Compound E binding is associated with a “closed” conformation.

The next steps for the field will be to build structural models of the complex associated with various interaction partners, inhibitors and modulators. These models will facilitate a mechanistic understanding of all intramembranous aspartyl proteases. Importantly, by mapping the binding site of different classes of GSM and GSI compounds, and by the defining the consequent three dimensional structural shifts in the architecture of the complex, it may be possible to design compounds targeting specific substrates and/or specific cleavage products.

## Abbreviations

PS1: Presenilin 1
PS2: Presenilin 2
APH1: Anterior pharynx 1
PEN-2: Presenilin enhancer 2
GSI: Gamma-secretase inhibitor compounds
GSM: Gamma-secretase modulator compounds
cryo-EM: Cryo- electron microscopy
SEC-MALS: Size exclusion chromatography multi angle light scattering
FRET FLIM: Förster resonance energy transfer fluorescence lifetime imaging
SPP: Signal peptide peptidase
MCMJR1: Clone name for Archeal presenilin-like homologue.
